# The Impact of Nutrient Solution Electrical Conductivity on Leaf Transcriptome Contributing to the Fruit Quality of Cucumber Grown in Coir Cultivation

**DOI:** 10.3390/ijms252211864

**Published:** 2024-11-05

**Authors:** Lizhong He, Wentao Xu, Dongke Zhou, Jun Yan, Haijun Jin, Hongmei Zhang, Jiawei Cui, Chen Miao, Yongxue Zhang, Qiang Zhou, Jizhu Yu, Xiang Yu, Xiaotao Ding

**Affiliations:** 1 Shanghai Key Laboratory of Protected Horticultural Technology, Horticultural Research Institute, Shanghai Academy of Agricultural Sciences, Shanghai 201403, China; helizhong@saas.sh.cn (L.H.); ylgw_07@126.com (J.Y.); jinhaijun@saas.sh.cn (H.J.); zhanghongmei@saas.sh.cn (H.Z.); cuijiawei@saas.sh.cn (J.C.); miaochen@saas.sh.cn (C.M.); zhangyongxue@saas.sh.cn (Y.Z.); zhouqiang@saas.sh.cn (Q.Z.); yujizhu@saas.sh.cn (J.Y.); 2 Joint International Research Laboratory of Metabolic & Developmental Sciences, School of Life Sciences and Biotechnology, Shanghai Jiao Tong University, Shanghai 200240, China; michaelwilliams@sjtu.edu.cn (W.X.); dongkezhou@sjtu.edu.cn (D.Z.)

**Keywords:** nutrient solution, electrical conductivity, coir cultivation, cucumber fruit quality, integrated omics analysis

## Abstract

Soilless cultivation is increasingly utilized in supplying essential nutrients for greenhouse crops. However, the impact of coir cultivation under varying electrical conductivity (EC) conditions on cucumber growth and fruit quality, particularly through the regulation of gene expression during the vegetative stage, remains uncertain. In this study, we performed metabolic measurements on cucumber in both vegetative and reproductive stages under three different EC conditions and found metabolic products such as some primary metabolites (cellulose, many uncharged amino acids) and some secondary metabolites (rutin, cucurbitacin B) accumulated the most under EC of 5 dS·m^−1^. Next, we conducted transcriptome profiling in cucumber leaves, revealing that the function of genes significantly regulated by EC was associated with photosynthesis, many anabolic processes, and membrane transport. Finally, a set of genes contributed to metabolites related to the fruit quality of cucumber were identified by the Orthogonal Partial Least Squares (O2PLS) analysis, including genes involved in the biosynthesis of amino acids, polysaccharides, and many secondary metabolites. Taken together, these findings suggest that coir cultivation in greenhouses with moderate EC can induce a transcriptome-wide change in gene expression, thereby contributing to enhancing the abundance of metabolites associated with cucumber fruit quality.

## 1. Introduction

Soilless cultivation of vegetables in controlled environments such as plastic or glass greenhouses has experienced substantial growth in recent years. This method allows for precise management of nutrient delivery and irrigation, optimizing the nutrient supply according to the vegetative cycle of the crop, offering advantages over traditional soil-based cultivation, and improving yield and product quality [1,2]. By circumventing soilborne pathogens and minimizing the risk of nitrate and pesticide leaching, soilless systems contribute to sustainable agricultural practices [3].

Cucumber, a prominent greenhouse crop, is notably susceptible to salinity stress [4]. The pH and electrical conductivity (EC) of the nutrient solution (NS) are usually monitored to evaluate the nutrient status and are critical determinants of plant growth, yield, and quality [5]. Discrepancies between nutrient supply and uptake can lead to the accumulation of mineral elements in the root zone substrate [6]. Elevated EC levels in the root environment create osmotic stress, impeding water uptake and potentially hindering plant development [7]. A suitable concentration of nutrient solution is very important for the growth, yield, and quality of cucumber [8].

To adapt to the changing external environments, plants produce various metabolic products, such as protein, amino acids, phenolics, alkaloids, etc. [9]. A growing body of evidence indicates that plant secondary metabolites are involved in plant stress defense and are beneficial for human health [10]. For example, drought stress could extensively induce phenolic, proline, soluble protein, lipid peroxidation, and flavonoid compounds in pepper plants [11,12]. A total of 80% of drought stress resulted in lettuce plants with higher levels of carotenoids, chlorophylls, caffeic acid, monocaffeoyl tartaric acid, malercyl quercetin glucoside, and greater total antioxidant activity at harvest [13]. The contents of proline and free amino acids in potatoes were also found to increase under drought stress [14].

In hydroponic systems, the EC of the nutrient solution is used as an index of nutrient salinity [15]. Cliff et al. reported that high EC (5 dS·m^−1^) in tomato cultivation resulted in softer, redder, and more flavorful fruits, albeit with reduced size [16]. Rodríguez et al. observed that a 4.5 dS·m^−1^ nutrient solution led to increased total soluble solids (TSS) and amino acid concentrations in fall–winter tomatoes, while spring–summer tomatoes exhibited higher TSS and organic acid levels [17]. Additionally, Lu et al. demonstrated that irrigating cherry tomato plants with a 4.5 dS·m^−1^ nutrient solution during fruiting and harvesting significantly improved plant height, net photosynthetic rate, and fruit quality [18]. Conversely, in sweet pepper, high EC levels negatively impacted plant growth parameters including plant height, stem diameter, shoot dry weight, and leaf net photosynthetic rate, while increasing oxidative stress markers such as malondialdehyde (MDA) and the activities of ascorbate peroxidase and guaiacol peroxidase [19].

Driven by economic and environmental considerations, growers are increasingly adopting sustainable and recyclable substrates like bark and coir as alternatives to rockwool and peat [20,21]. Xing et al. found that the EC in root-zone solution reached 7.71 dS·m^−1^ in coir, 5.86 dS·m^−1^ in peat–vermiculite, and 4.12 dS·m^−1^ in water culture, where tomato plants were grown in a semicircle without any substrate. The coir cultivations led to higher concentrations of K^+^, NO_3_^−^, SO_4_^2−^, and Mg^2+^ in the tomato root-zone solution and induced complex proteomic alterations related to mineral ion transport [22]. Our previous study also revealed that coir significantly enhanced cucumber leaf area index (LAI), yield, and the accumulation of Ca, Mg, S, Cl, and Zn in leaves and fruits compared to rockwool. Furthermore, coir positively influenced the amino acid and flavor compound profiles of cucumber fruits [23].

In cucumber, the effects of coir-based soilless cultivation with varying levels of EC on transcriptome-wide gene expression during vegetative growth and on fruit quality-associated metabolites remain poorly understood. To address this knowledge gap, we investigated the influence of different nutrient solution concentrations on fruit quality, photosynthesis, and leaf transcriptomic profiles in coir-grown cucumbers. Our findings reveal a suite of genes significantly regulated by high EC levels, potentially contributing to the biosynthesis of metabolites related to cucumber fruit quality. These findings offer insight into the expression and regulation of genes associated with fruit quality and osmotic stress, providing crucial information for optimizing the cost-efficiency and sustainability of coir-based cultivation systems in modern greenhouses.

## 2. Results

### 2.1. Biochemical Parameters of Cucumber Growth Under Different EC Conditions

To explore the effects of varying electrical conductivity (EC) levels of the nutrient solution (NS) on seedling growth and fruit quality of cucumber in coir cultivation, we measured intercellular CO_2_ concentration (Ci), stomatal conductance (Gs), the net photosynthesis rate (Pn), water use efficiency (Wue), and chlorophyll content (both Chl A and Chl B). From this analysis, we found that Ci and Gs were relatively stable at lower EC levels but exhibited a sharp increase at the highest EC level (CL8). Interestingly, the net photosynthesis rate (Pn), water use efficiency (Wue), and chlorophyll content (both Chl A and Chl B) displayed a declining trend with increasing EC of the NS.

**Figure 1:**
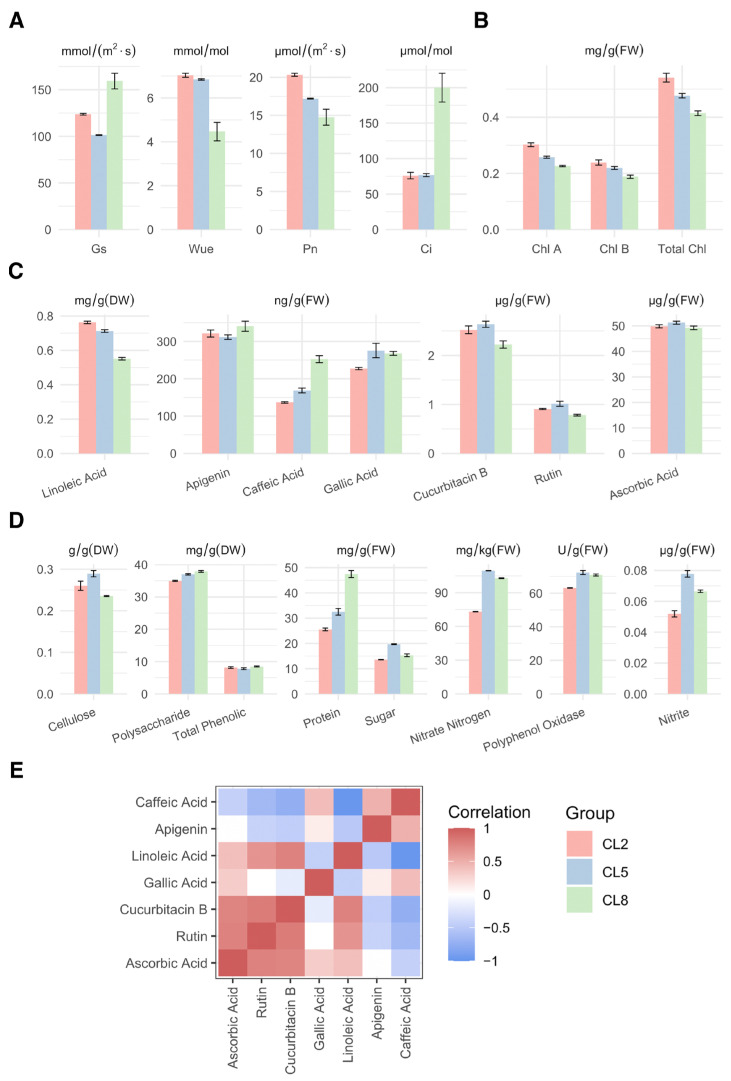
Physiological characteristics and abundance of metabolites of *Cucumis sativa* under different electrical conductivities of nutrient solutions, denoted in fresh weight (FW) or dry weight (DW). (**A**) Quantification of intercellular CO_2_ concentration (Ci), stomatal conductance (Gs), the net photosynthesis rate (Pn), and water use efficiency (Wue). (**B**) Abundance of chlorophyll A (Chl A), chlorophyll B (Chl B), and total chlorophylls. (**C**) Abundance of secondary metabolites. (**D**) Abundance of some primary metabolites, enzymes, and other substances indicative of plant overall metabolism. Content of protein is determined using bicinchoninic acid assay. (**E**) Pairwise correlation coefficient of the abundance of secondary metabolites.

The abundance of secondary metabolites (Figure 1C) and primary metabolites (Figure 1D) generally peaked in the CL5 group, suggesting an optimal level of nutrient availability, while the CL8 group exhibited suboptimal levels. This observation indicates that plants may rely less on photosynthesis for their metabolic processes when an appropriate level of nutrients is available (in this case, CL5) in the culture solution. Notably, the protein content (Figure 1D) and amino acid levels (Figure S1A) significantly increased in the CL8 group compared to CL5, indicating that higher EC in the nutrient solution may boost protein and amino acid synthesis, potentially as a stress response mechanism.

Pearson’s correlation analysis (Figure 1E) revealed that among the secondary metabolites examined, ascorbic acid, rutin, cucurbitacin B, and linoleic acid exhibited positive correlations in their abundance, while other secondary metabolites showed less significant relationships. Additionally, the abundance of all 17 amino acids examined and primary metabolites (Figure S1B) appeared to be significantly positively correlated, with which chlorophylls were significantly negatively related. These findings suggest that an appropriate EC level in the NS may promote metabolite synthesis in cucumber while reducing reliance on photosynthesis. However, excessively high EC levels (as in CL8) appear to induce stress responses, such as increased protein and amino acid synthesis, potentially as a protective mechanism against osmotic stress.

### 2.2. Identification of Genes Regulated by High EC Conditions

**Figure 2:**
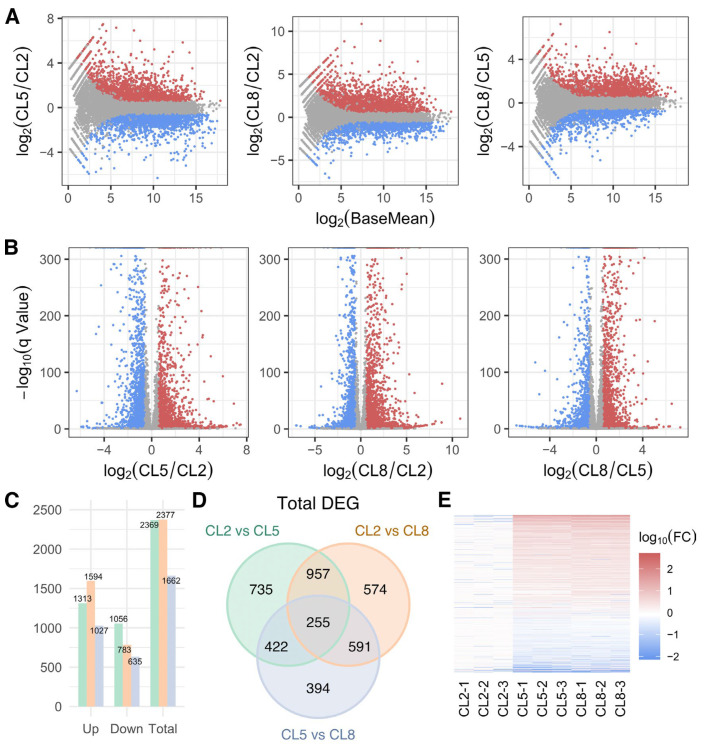
Statistics of differentially expressed genes (DEG) in each pair of groups. (**A**,**B**) MA plot and volcano plot of DEG fold changes. Base Mean and q value are calculated using DEseq2. Colored dots indicate |log_2_ (fold change)| > 0.58 and q-Value < 0.05. (**C**) Statistics of up- or downregulated DEG. (**D**) Venn diagram showing distribution of DEG. Numbers in black indicate number of DEG within the corresponding part of diagram. DEGs referred to in the following analysis are the union of DEGs in this diagram. (**E**) Overview of DEG expression level changes. Numbers to the color bar represent log_10_(fold change). Each row corresponds to one gene and each column represents one repetition in a group. Fold changes are calculated using average of group CL2 of each row as reference.

To investigate transcriptional changes in cucumber plants in response to varying EC levels of the nutrient solution, transcriptome profiling was performed on cucumber leaves for all three replicates under the three different NS concentrations. From this analysis, a substantial number of genes exhibited significant differential expression across these different conditions (Figure 2A,B). Compared to CL2, 2369 and 2377 genes were significantly altered under CL5 and CL8, respectively (Figure 2C), while 1662 differentially expressed genes were identified between CL5 and CL8. These findings indicate that changes in EC levels profoundly influence gene expression in cucumber plants. Generally, more genes were upregulated than downregulated across all comparisons (Figure 2C,E). Compared to CL2, 1313 and 1594 genes were upregulated in CL5 and CL8, respectively, while 1056 and 783 genes were downregulated. This pattern suggests that the plants activate a broad range of genetic pathways to cope with increased EC levels. A total of 1212 genes were significantly changed in both CL5 and CL8 compared to CL2 (Figure 2D). Specifically, compared to CL2, the abundance of 451 genes was increased, while the abundance of 719 genes was decreased in both CL5 and CL8 (Figure S2). These commonly affected genes likely play crucial roles in the plant’s response to varying EC levels. Taken together, the substantial remodeling of gene expression patterns under high EC conditions suggests that cucumber plants undergo extensive transcriptional reprogramming to adapt to changes in nutrient availability.

### 2.3. Functional Analysis of DEGs Reveals Enhanced Metabolism and Stress Response

**Figure 3:**
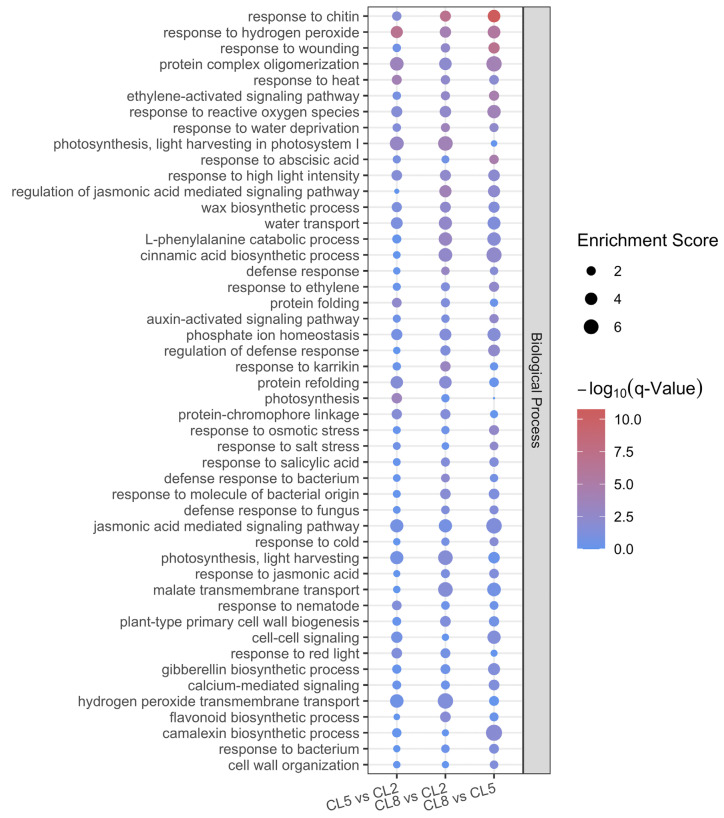
GO enrichment analysis of DEG. Top enriched GO terms in biological process with FDR < 0.05, ranked by log_10_(FDR).

To gain insights into the biological processes, cellular components, and molecular functions associated with the transcriptional responses to varying electrical conductivity (EC) levels of the nutrient solution (NS), we performed Gene Ontology (GO) enrichment analysis for the differentially expressed genes (DEGs) in each comparison (Figure 3 and Figure S3). The enrichment analysis revealed that plant metabolism is enhanced with increasing EC levels in the NS, as evidenced by the enrichment of terms such as plant-type primary cell wall biogenesis, protein folding, peptide transmembrane transporter activity, flavonoid biosynthetic process, camalexin biosynthetic process, acyl transferase activity, and UDP-glucosyltransferase activity. Cellular respiration also appears to be greatly enhanced, as shown by the enrichment of the term malate transmembrane transport.

Interestingly, photosynthesis activity, represented by terms like photosystem I, photosystem II, thylakoid, chlorophyll binding, and photosynthesis, displays an increase-then-decrease trend. Combining this observation with the previously discussed changes in biochemical parameters, we can conclude that the optimal EC condition for plant photosynthesis lies around the CL5 group, while excessively high EC levels in the CL8 group depress photosynthetic activities.

However, the plants appear to be under stress as EC increases, as evidenced by the enrichment of stress-related terms. Responses to osmotic pressure, including response to water deprivation, water transport, response to salt stress, and water channel activity, are significantly enriched as EC increases. Responses to other abiotic stresses, such as oxidative stress (e.g., response to hydrogen peroxide, reactive oxygen species), abscisic acid signaling, and ethylene signaling, are also enriched, suggesting the activation of various stress response pathways.

Notably, the enrichment of biotic stress responses, including response to chitin, bacterium, and fungus, highlights the possibility of suitable environments for microbial growth when EC levels in the nutrient solution are relatively high. Taken together, the GO enrichment analysis of DEGs highlights that increasing electrical conductivity levels in the nutrient solution stimulate plant metabolism and cellular respiration, while also inducing stress responses, particularly to osmotic and biotic stresses.

### 2.4. Integrated Transcriptomic and Metabolomic Analysis Reveals Potential Associations Between Genes and Metabolites

To identify potential associations between genes and metabolites that could mediate the observed changes induced by varying electrical conductivity (EC) levels in the nutrient solution, we performed an integrated analysis of the transcriptomic and metabolomic data using the Orthogonal Two-way Partial Least Squares (O2PLS) method. This multivariate approach decomposes the variation in two datasets into joint, dataset-specific, and noise components, enabling the identification of correlations between the datasets while accounting for their intrinsic structures.

**Figure 4:**
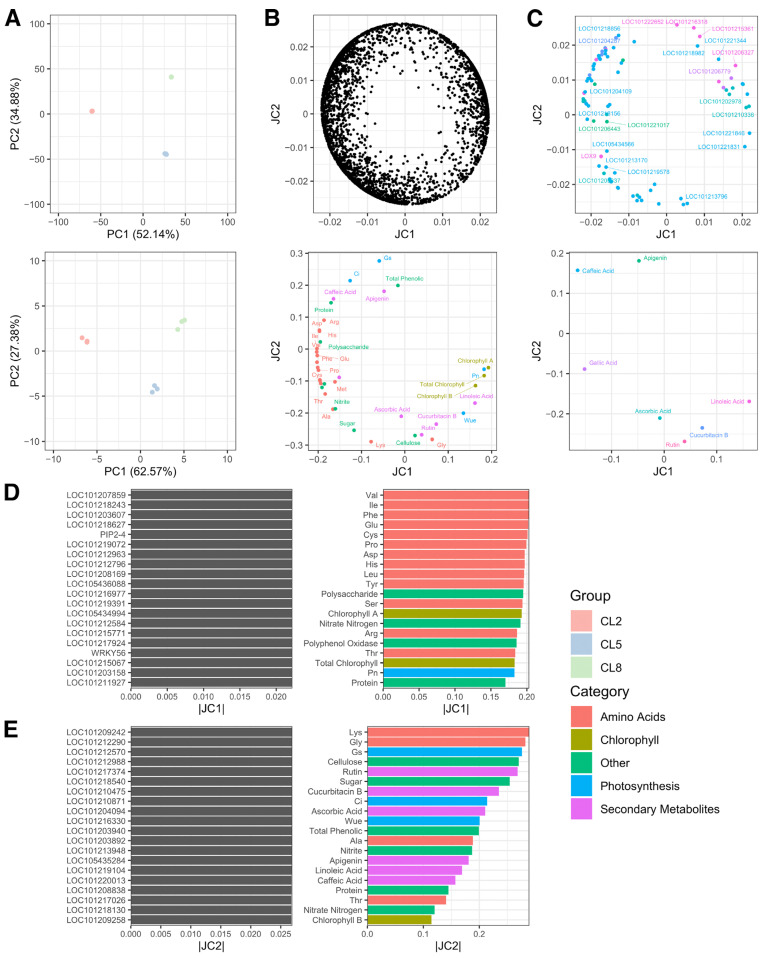
PCA and O2PLS analysis of metabolomic and transcriptomic data. (**A**) Principal Component Analysis of transcriptomic data (upper panel) and metabolomic data (lower panel). Both show good consistency of repetitions within a group. (**B**) O2PLS analysis of transcriptomic (upper panel) and metabolomic (lower panel) data integrated. Groups of genes with high absolute values on a component are hypothetically related to groups of metabolites with high absolute values on the same component axes. JC, joint component. (**C**) Selected data points of the O2PLS analysis results. Upper panel shows transcriptomic data points of genes related to secondary metabolites in the lower panel according to KEGG database. A metabolite and its related genes share the same color across the two panels. (**D**,**E**) Top 20 items ranked by absolute joint component values in the above O2PLS analysis.

Principal Component Analysis (PCA) revealed high consistency within replicates of each group and distinct separation among different EC groups, indicating high data quality and reproducibility (Figure 4A). This foundation allowed for a robust O2PLS analysis. The O2PLS model (Figure 4B) demonstrated a relatively clear distinction among amino acids, photosynthesis, and chlorophyll categories on the joint components 1 and 2. However, primary and secondary metabolites were not well resolved, potentially due to the diverse and unrelated metabolic pathways and functions represented within these broader categories. The transcriptome data, comprising approximately 3000 differentially expressed genes (DEGs) across three groups with three replicates each, exhibited a more complex structure that was not clearly resolved on the joint components (Figure 4B, upper panel).

Focusing specifically on secondary metabolites (Figure 4C, lower panel), we observed a certain degree of relatedness among ascorbic acid, cucurbitacin B, and rutin, corroborating the correlation analysis in Figure 1E and their known metabolic pathway relationships according to the KEGG database. We also selected genes related to the metabolism of these secondary metabolites (Figure 4C, upper panel); however, no significant associations were evident, suggesting that additional factors or regulatory mechanisms may be involved.

Using the O2PLS model, we identified groups of related genes and metabolites on joint component 1, which appeared to be primarily associated with amino acid metabolism (Figure 4D), and joint component 2, which had a greater emphasis on primary and secondary metabolites (Figure 4E). These findings suggest that different components of the integrated model capture distinct aspects of the transcriptional and metabolic responses to varying EC levels, potentially reflecting the underlying biological processes and pathways involved.

**Figure 5:**
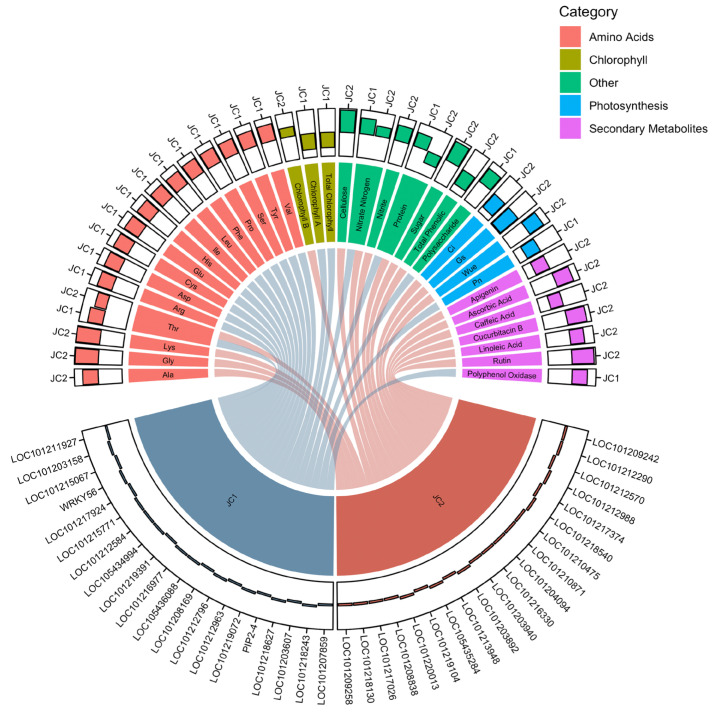
Summary of related groups of genes and metabolites predicted by O2PLS. Bars extending inwards indicate positive value of the component.

The identified relationships between groups of genes and groups of metabolites are visually represented in Figure 5, providing a concise overview of the potential associations revealed by the integrated analysis. It is important to note that these associations do not necessarily imply direct causal relationships but rather highlight potential co-regulation or co-occurrence patterns that warrant further investigation.

### 2.5. Anabolism of Secondary Metabolites Is Significantly Influenced by EC of Nutrient Solutions

**Figure 6:**
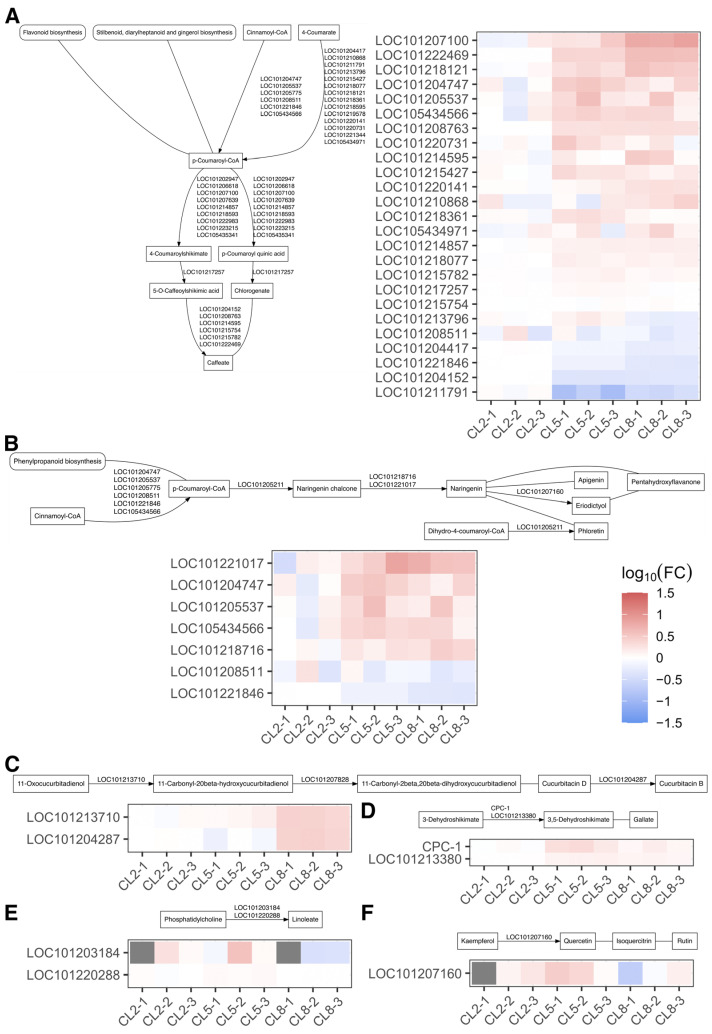
Expression level of DEGs directly involved in the synthesis of some secondary metabolites, (**A**) caffeic acid, (**B**) apigenin, (**C**) cucurbitacin b, (**D**) gallic acid, (**E**) linoleic acid, and (**F**) rutin. Pathway data are retrieved from KEGG database. Box represents compound and rounded box represents pathway. Line with no arrowhead denotes that the reaction is not reported in *Cucumis sativa* but homologous enzyme of this reaction in other organisms has been discovered.

**Figure 7:**
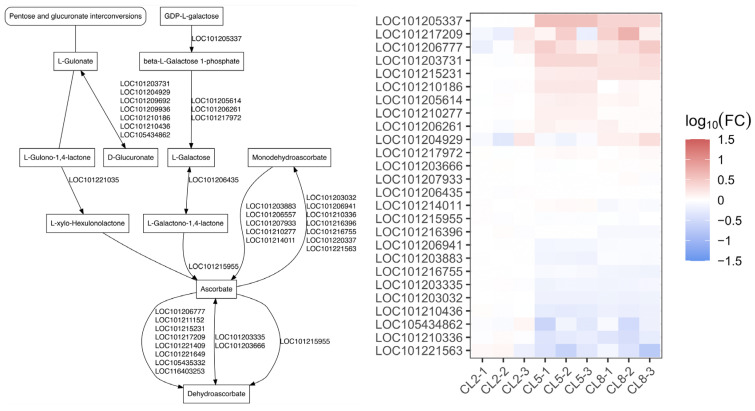
Expression level of DEGs directly involved in the synthesis of ascorbic acid.

Finally, we acquired pathway data from the KEGG database on the anabolism steps four steps upstream of the secondary metabolites examined in this research. We identified genes in our transcriptome data corresponding to enzymes in the synthetic pathways related to caffeic acid, linoleic acid, apigenin, gallic acid cucurbitacin B, and rutin, and found that high EC induced significant changes in the expression levels of these genes (Figure 6 and Figure 7). Most of the enzymes functioning in synthetic pathways of caffeate were induced by high EC (Figure 6A), consistent with the increase in caffeic acid abundance in CL5 (EC = 5) and CL8 (EC = 8) compared to CL2 (EC = 2) (Figure 1C). However, the induction of genes involved in cucurbitacin B biosynthesis contrasted with the decrease in cucurbitacin B in EC8 (Figure 1C and Figure 6C), indicating that the gene expression pattern may differ between leaf and fruit. *CPC-1* and *LOC101213380* involved in gallate synthesis were also induced by high EC (Figure 6D), and gallic acid also increased under high EC conditions (Figure 1C). Additionally, *LOC101203184* was specifically downregulated in EC8 (Figure 6E), consistent with the dramatic decrease in linoleic acid in EC8 condition (Figure 1C). The genes related to ascorbate showed diverse changes under high EC, while ascorbic acid displayed robust abundance during the three EC conditions (Figure 7). Taken together, the high EC conditions induced substantial changes in the expression levels of genes associated with the biosynthesis of key secondary metabolites.

## 3. Discussion

The abiotic stresses, such as salinity, drought, heat, and cold, have various detrimental effects on plant physiology, including growth, biomass accumulation, productivity, seed production, and fruit quality [24]. Our investigation of the effects of varying electrical conductivity (EC) levels of nutrient solutions (NS) on fruit quality of cucumber growing in coir cultivation, reveals several key insights into the physiological and biochemical responses of the plants. Measurements of intercellular CO_2_ concentration (Ci) and stomatal conductance (Gs) displayed stability at lower EC levels, with a significant increase at the highest EC level (CL8) (Figure 1A). This sharp rise is indicative of stress-induced stomatal responses aimed at mitigating potential damage from high salt concentrations. Despite stable Ci and Gs at lower EC levels, net photosynthetic rate (Pn), water use efficiency (Wue), and chlorophyll content (both Chl A and Chl B) showed a declining trend with increasing EC (Figure 1A,B). This decline suggests that higher EC levels may inhibit the photosynthetic machinery, leading to reduced efficiency in water use and chlorophyll synthesis, which are critical for plant growth and productivity. The osmotic stress and ion imbalance, induced by salt stress, could also decrease the photosynthetic rate, enzymatic activity, and chlorophyll content [25] while facilitating the accumulation of reactive oxygen species (ROS) [26]. Interestingly, our results show that secondary and primary metabolites peaked in the CL5 group (Figure 1C,D), suggesting that moderately increasing the EC level of NS is beneficial for plant nutrient absorption. This optimal level likely provides a balanced environment that supports metabolic processes without imposing overwhelming stress, thereby enhancing metabolite synthesis. In contrast, the synthesis of these metabolites was significantly inhibited in the CL8 group, further supporting the notion that excessively high EC levels are detrimental to overall plant metabolism. Pearson’s correlation analysis provided additional insight into the relationships between various metabolites. Secondary metabolites such as ascorbic acid, rutin, cucurbitacin B, and linoleic acid showed positive correlations in their abundance (Figure 1E), suggesting a coordinated metabolic regulation under varying EC levels. On the other hand, the significant positive correlation between all 17 amino acids examined, along with primary metabolites, underscores the intricate link between nutrient availability and metabolic processes (Figure S1B). The synthesis and accumulation of osmotic solutes in the cytoplasm, such as soluble protein, sugars, and amino acids (Figure 1D and Figure S1A), may serve to maintain cellular homeostasis and osmotic equilibrium, protecting cellular structures against osmotic stress induced by high EC of NS [27]. In addition, our results also showed that the high EC had a negative effect on photosynthesis and chlorophyll content (Figure 1A,B). This suggests that as plants prioritize metabolite synthesis, particularly under stress conditions (e.g., high EC), there is a concomitant decline in chlorophyll content, possibly due to resource reallocation. Xu and Mou reported that mild salt stress (EC = 4.6–6.5 dS·m^−1^) could enhance flavonoid, total phenolic content, protein content, and amino acid content of spinach and 40 mM NaCl treatment (EC > 10.2 dS·m^−1^) caused 50% reduction in fresh shoot weight of spinach [28]. Similarly, in tomatoes, soluble solids, carbohydrates, sodium, and chloride concentrations increased as water salinity increased from 0.5 dS·m^−1^ to 15.7 dS·m^−1^. Meanwhile, total carotenoids and lycopene concentrations peaked at an EC of 4.4 dS·m^−1^ and then declined with further increases in salinity [29]. As for cucumber, NaCl stress (EC = 4.9 and 6.8 dS·m^−1^) could improve fruit quality by increasing fruit dry matter, soluble sugar, and titratable acidity [30]. In this research, the protein content, sugar, amino acid levels, and some secondary metabolites were also found significantly higher in groups CL5 and CL8 (Figure 1 and Figure S1). This increase likely represents intracellular water loss induced by hyperosmotic stress, which affects protein dynamics and protein–protein interactions [31]. It is important to note that nutrient solution concentrations for greenhouse cucumber production are generally maintained around 3 dS·m^−1^, high EC may increase cucumber fruit quality, but also negatively affects total yield [30].

As EC levels increase, the plants show signs of stress, as evidenced by the enrichment of stress-related GO terms (Figure 3). Osmotic stress responses, such as response to water deprivation, water transport, response to salt stress, and water channel activity, are significantly enriched, indicating that the high EC may cause hyperosmotic stress to plants. Hyperosmotic stress induces efflux of water from the cell, and plants actively manage water balance and osmotic pressure to increase intracellular water content [31]. The enrichment of responses to oxidative stress, including responses to hydrogen peroxide and reactive oxygen species, as well as abscisic acid and ethylene signaling pathways, further suggests the activation of various stress response mechanisms aimed at protecting plants from adverse environmental conditions. Notably, the enrichment of GO terms related to biotic stress responses, such as response to chitin, bacterium, and fungus, highlights the potential for increased microbial activity in high EC environments. The GO enrichment analysis underscores the multifaceted impact of EC levels on cucumber plants. While moderately high EC levels (e.g., CL5) enhance plant metabolism and photosynthetic efficiency, excessively high EC levels (e.g., CL8) induce significant abiotic and biotic stress responses. These findings highlight the delicate balance required in managing nutrient solution EC to optimize plant growth and health. Excessive EC can lead to osmotic stress, reduced photosynthetic efficiency, and increased susceptibility to microbial infection, ultimately affecting overall plant productivity.

These findings underscore the importance of optimizing EC levels in nutrient solutions for cucumber cultivation. Appropriately elevated EC levels in nutrient solutions (such as CL5) promote metabolite synthesis and maintain overall plant health, while excessively high EC levels (such as CL8) induce stress responses that can hamper growth and productivity. Previous research on leaf lettuce has shown that nutrient solution deprivation can reduce the yield and some quality indexes, such as color parameters and anthocyanin concentration [32]. Meanwhile, excessive nutrient concentration can have adverse effects on lettuce yield and leaf nitrate of lettuce due to the high osmotic pressure around the root [33]. High soil EC could also decrease plant K^+^ concentration, chlorophyll B, carotenoid, biomass, and fruit quality of cucumber [34]. Therefore, careful management of nutrient solution EC is crucial for achieving optimal plant performance and maximizing yield and fruit quality. Further research is warranted to explore the underlying mechanisms of these responses and to identify strategies to mitigate the adverse effects of high EC levels in horticultural practices.

Next, by analyzing the transcriptional changes, we identified numerous differentially expressed genes (DEGs) that highlight the complexity of the plant’s adaptive strategies to different nutrient availabilities (Figure 2). The analysis showed a general trend of more genes being upregulated than downregulated across all comparisons. The Gene Ontology (GO) enrichment analysis sheds light on how different EC levels modulate plant metabolism, photosynthesis, and stress response pathways (Figure 3). The enrichment of GO terms such as plant-type primary cell wall biogenesis, protein folding, peptide transmembrane transporter activity, flavonoid biosynthetic process, camalexin biosynthetic process, acyl transferase activity, and UDP-glucosyltransferase activity indicates a significant enhancement in plant metabolism with increasing EC levels. This suggests that higher EC levels may boost metabolic activities, potentially leading to increased synthesis of structural components and secondary metabolites. It has been reported that plant osmosensors perceive changes in plasma membrane tension, the integrity of the cell wall–plasma membrane interface, extracellular osmolarity, and the concentration of macro-molecules inside the cell [35]. The hyperosmotic stress induced by high EC led to a reduction in cell volume and an increase in solute concentrations and also affected protein structure, protein dynamics, and protein–protein interactions [31]. Additionally, the enrichment of malate transmembrane transport suggests a notable enhancement in cellular respiration, which is crucial for energy production and metabolic processes. Interestingly, the GO terms related to photosynthesis, such as photosystem I, photosystem II, thylakoid, chlorophyll binding, and photosynthesis, exhibited an increasing-then-decreasing trend. This aligns with earlier observations from the biochemical parameters, indicating that the optimal EC level for photosynthesis is around the CL5 group. Excessively high EC levels in the CL8 group appear to depress photosynthetic activities, likely due to osmotic stress and resource reallocation towards stress mitigation.

The integrated analysis of transcriptome and metabolome data using the Orthogonal Two-way Partial Least Squares (O2PLS) method enabled us to identify potential associations between genes and metabolites that may mediate the observed physiological responses. The O2PLS model identified groups of related genes and metabolites on joint component 1, primarily associated with amino acid metabolism, and joint component 2, which emphasized primary and secondary metabolites (Figure 4). These findings suggest that the integrated model captures distinct aspects of the transcriptional and metabolic responses to varying EC levels. The integrated analysis underscores the importance of considering both transcriptional and metabolic data to gain a comprehensive understanding of plant responses to environmental changes. The identified associations between genes and metabolites suggest potential pathways and regulatory networks involved in adapting to varying EC levels. For example, proline is considered a compatible osmolyte and can be used for osmotic adjustment as well as protection against oxidative stress [36]. In tomatoes, compatible osmolytes such as glycine and proline were reported to accumulate significantly under osmotic stress and excessive ROS [37]. Future research should focus on elucidating these pathways and regulatory mechanisms, potentially through functional genomics and targeted metabolomics approaches. By integrating transcriptomic and metabolomic data, this study provides a holistic view of the cucumber plant’s responses to nutrient solution EC levels. This approach can be applied to other crops and environmental conditions to better understand plant adaptation and improve agricultural practices.

Finally, our findings indicate that high EC conditions induce significant changes in the expression levels of genes associated with the biosynthesis of several key secondary metabolites, such as caffeic acid, cucurbitacin B, gallic acid, linoleic acid, and ascorbic acid (Figure 6 and Figure 7). The upregulation of specific biosynthetic genes suggests enhanced production of certain metabolites, while discrepancies between gene expression and metabolite abundance in some cases point to complex, tissue-specific regulatory mechanisms. These findings provide valuable insights into how cucumber plants adapt their metabolic networks in response to nutrient availability, offering potential strategies for optimizing plant growth and stress tolerance through nutrient management. Further research, including tissue-specific expression analyses and functional studies, will be essential to fully unravel these regulatory pathways and their implications for cucumber cultivation.

## 4. Materials and Methods

### 4.1. Plant Material and Growing Conditions in Coir Cube

The Cucumber (*Cucumis sativus*) cultivated variety Deltastar (Rijk Zwaan Distribution B.V., De Lier, The Netherlands) was utilized in this study. Briefly, cucumber seeds were sown and germinated in coir cubes (10 cm × 10 cm × 6.5 cm, 100% 0–6 mm coir, EC < 1 dS·m^−1^, pH 5.8–6.8, Remmy, Qingdao Remmy Commerce and Trade Co., Ltd., Qingdao, China) under natural light with a maximum photosynthesis photon flux density 1200 μmol m^−2^ s^−1^. Daytime and nighttime temperatures were maintained at 25 ± 2 °C (day) and 17 ± 2 °C (night), respectively. The cucumber seedlings were regularly irrigated using a modified Hoagland nutrient solution (pH = 5.5, EC = 2.0 dS·m^−1^). Once the plants reached the 7–8 leaf stage, the cucumber seedlings were transferred to coir slabs (100 cm × 20 cm × 8 cm, 50% 0–6 mm coir, 50% 10–20 mm coir, EC < 1, pH 5.8–6.8). The plants were irrigated with nutrient solutions of varying concentrations (EC = 2 dS·m^−1^ (CL2), 5 dS·m^−1^ (CL5), and 8 dS·m^−1^ (CL8)) through an automatic irrigation system (Nutrjet 300 inline, Priva B. V., De Lier, The Netherlands) with one drip of 100 mL every 80–100 J cm^−2^ of radiation sum per plant per day. The irrigation volume and frequency were consistent across all slabs, with drainage maintained at around 20% daily. The irrigation system comprised the following two tanks: the A Tank (500 L) contains Ca(NO_3_)_2_ 87.5 kg, EDTA-Fe 750 g, and the B Tank (500 L) contains KNO_3_ 25 kg, MgSO_4_ 30 kg, KH_2_PO_4_ 12 kg, K_2_SO_4_ 3 kg, Na_2_B_4_O_7_ 90 g, MnSO_4_ 120 g, ZnSO_4_ 62 g, CuSO_4_ 13 g, and Na_2_MoO_4_ 8 g.

### 4.2. Library Construction of Transcriptome

Total RNA was isolated from leaves of *Cucumis sativa* samples using TRIzol reagent (Invitrogen, Carlsbad, CA, USA) according to the manufacturer’s protocol. RNA purity and integrity were assessed using a NanoDrop 2000 spectrophotometer (Thermo Fisher Scientific, Waltham, Massachusetts, USA) and an Agilent 2100 Bioanalyzer (Agilent Technologies, Santa Clara, California, USA), respectively. RNA-seq libraries were prepared using the VAHTS Universal V6 RNA-seq Library Prep Kit (Vazyme Biotech, Nanjing, China) following the manufacturer’s instructions. Paired-end sequencing (150 bp) was performed on an Illumina Novaseq 6000 platform, generating approximately 45–50 million raw reads per sample (OE Biotech Co., Ltd., Shanghai, China).

### 4.3. Analysis of Transcriptome Data

Raw reads were quality-filtered using fastp [38], retaining 44–50 million clean reads per sample. Clean reads were aligned to the *Cucumis sativa* reference genome (Cucumber_9930_V3) using HISAT2 [39]. The raw read count per gene was calculated using HTSeq-count [40]. Gene expression levels were normalized as fragments per kilobase of transcript per million mapped reads (FPKM) [41]. Differential gene expression analysis was conducted using DESeq2 [42], with significantly differentially expressed genes (DEGs) determined by a q-value < 0.05 and a fold change > 2 or < 0.5. Visualization of DEG distributions among various groups was accomplished through Venn diagrams, while bar charts illustrated the up- or downregulation of DEGs within each group comparison. Normalized expression levels of DEGs were presented as heat maps relative to the CL2 group. Gene Ontology (GO) enrichment analysis was conducted for pairwise DEGs and their union set [43,44,45]. Additionally, the genes involved in secondary metabolite biosynthesis were examined based on the KEGG pathway [46,47,48] and depicted as heat maps. Volcano and MA plots were generated to assess the significance and magnitude of differential expression in each comparison.

### 4.4. Quantification of Biochemical Parameters in Leaves and Fruit of Cucumbers

Biochemical parameters are measured with the same method as in our previous study [23]. All analyses were performed in triplicate (*n* = 3).

Briefly, photosynthetic parameters including net photosynthetic rate (Pn), stomatal conductance (Gs), and intercellular CO_2_ concentration (Ci) were measured using a portable photosynthesis system (CIRAS-3, PP Systems, Amesbury, MA, USA) equipped with a leaf chamber fluorometer (PLC3 Universal Leaf Cuvette, 18 by 25 mm window, CFM-3).

Free amino acid content was determined following the same method described in our previous study. Briefly, fruit samples (0.5 g) were homogenized in ultrapure water and derivatized using phenyl isothiocyanate. Derivatized amino acids were separated using a high-performance liquid chromatography system (Rigol L3000, Rigol Technologies Co., Ltd., Suzhou, China) equipped with a reverse-phase column (Sepax C18, 250 mm × 4.6 mm, 5 μm). Amino acid content was quantified based on peak areas and expressed as μg amino acid per gram fresh weight.

Secondary metabolites, including gallic acid, caffeic acid, apigenin, rutin, and cucurbitacin B, were quantified using high-performance liquid chromatography (HPLC) following the method described by Canas et al. and Mi et al. [49,50]. Linoleic acid was analyzed by gas chromatography after acid-catalyzed transmethylation, according to Moretti et al. [51].

Fruit-soluble protein content was quantified according to the Bradford method [52].

Polyphenol oxidase (PPO) activity was assessed as described by Tang and Newton [53]. Briefly, fruit tissue was homogenized in extraction buffer (100 mM NaPO_4_, pH 7.2, 0.1% [*w*/*v*] SDS, 3 mM ascorbate) and centrifuged. Supernatant PPO activity was measured spectrophotometrically at 490 nm and 28 °C, monitoring the conversion of L-dihydroxyphenylalanine (L-DOPA) to quinone polymers. Enzyme activity was expressed as the change in absorbance per milligram protein per minute (U), with 0.05 absorbance change defined as one unit.

Soluble sugar content was determined using the method of Bai et al. [54]. Briefly, 0.05 g of fruit tissue was boiled in 6 mL of deionized water for 30 min, centrifuged at 12,000× *g* for 10 min, and the supernatant was collected. The extract was diluted to 50 mL with deionized water, and 0.1 mL was reacted with 3 mL of anthrone reagent (0.15 g anthrone in 84 mL sulfuric acid and 16 mL water). Absorbance was measured at 620 nm.

Total polysaccharide (TP) content was determined using the phenol-sulfuric acid method [55]. Briefly, dried, ground fruit samples were mixed with distilled water (1:10 *w*/*v*) and extracted three times at 100 °C for 3 h. The filtered extract was precipitated with cold ethanol (8 °C) at a 4:1 volume ratio, refrigerated for 48 h at 4 °C, and centrifuged at 5000× *g* for 10 min. The polysaccharide content was then determined by the phenol-sulfuric acid method.

Ascorbate (AsA) and dehydroascorbate (DHA) contents were determined as described by Ali et al. [56]. AsA was measured by the decrease in absorbance at 265 nm after the addition of ascorbate oxidase (0.01 units/mL). DHA was measured by the increase in absorbance at 265 nm after the addition of dithiothreitol (DTT). Total AsA concentration was calculated as the sum of AsA and DHA.

Total chlorophyll content was determined spectrophotometrically according to Chung et al. [57]. Absorbance was measured at 663 nm, 645 nm, and 470 nm, and chlorophyll a, chlorophyll b, and total chlorophyll content were calculated using the following equations:

$$\mathrm{Chlorophyll}\;a=\frac{0.01\times \left(12.7\times A_{663}-2.69\times A_{645}\right)}{FW}$$

$$\mathrm{Chlorophyll}\;b=\frac{0.01\times \left(22.9\times A_{645}-4.68\times A_{663}\right)}{FW}\;$$

$$\mathrm{Total}\;\mathrm{Chlorophylls}=\frac{0.01\times \left(8.02\times A_{663}+20.21\times A_{645}\right)}{FW}$$

Total phenol content was determined using the Folin–Ciocalteu method [58]. Briefly, 5 mL of extract was mixed with 180 μL of distilled water and 1200 μL of Folin–Ciocalteu reagent (10%). After 5 min, sodium carbonate (7.5%) was added, and absorbance was measured at 760 nm. Total phenol content was expressed as mg of gallic acid equivalents (GAE) per gram of dry weight (DW).

Nitrate nitrogen (NO_3_-N) and nitrite (NO_2_-N) contents were determined spectrophotometrically according to Qiao et al. [59], and cellulose content was determined according to Tong et al. [60].

### 4.5. Analysis of Metabolite Data

Pearson’s correlation coefficients were calculated between all pairs of metabolites. Metabolites were categorized into distinct groups, such as amino acids, photosynthesis, chlorophylls, secondary metabolites, and others, based on their identities. The correlation matrix was sorted in descending order of row means within each category. Heatmaps were generated to visualize the correlation patterns among all metabolites and within the secondary metabolite category. Highly correlated metabolite pairs (absolute correlation ≥ 0.95) were identified. Bar plots were constructed to display the mean values and standard errors of metabolite concentrations across different groups. Finally, pairwise *t*-tests were conducted to compare metabolite levels between groups. The resulting *p*-values were used for the identification of significantly differentially abundant metabolites.

### 4.6. Integrated Omics Analysis

Principal Component Analysis (PCA) was conducted to assess the reproducibility and consistency of transcriptome and metabolite data within each group. Subsequently, Orthogonal Partial Least Squares (O2PLS) analysis was employed to integrate the transcriptome and metabolite data. O2PLS is a multivariate data integration technique that decomposes the variation in two datasets into joint, dataset-specific, and noise components, enabling the identification of correlations between the datasets while accounting for their intrinsic structure [61].

Prior to analysis, the data were centered around zero and scaled. To determine the optimal number of components, cross-validation was performed using the ‘crossval_o2m_adjR2()’ function, which adjusts for cross-validation in O2PLS analysis. This process yielded values for ‘*n*’ (number of joint components), ‘nx’ (number of transcriptome-specific components), and ‘ny’ (number of metabolite-specific components). In this study, ‘*n* = 3’, ‘nx = 0’, and ‘ny = 1’ were selected to best fit the omics data to the O2PLS model.

In the O2PLS model, the joint components capture the covariance between the transcriptome and metabolite data, while the dataset-specific components capture the variation unique to each dataset. The loading values for each variable (gene or metabolite) on the joint components indicate their relative importance in determining the joint variation. Variables with high loading values on the same joint component are strongly correlated. Therefore, by examining the variables with high loading values on the joint components, it is possible to identify groups of genes and metabolites that are related, potentially reflecting underlying biological processes or pathways.

The joint components were visualized by plotting differentially expressed genes (DEGs) and metabolites on respective panels, facilitating visual representation and interpretation similar to PCA results. To focus specifically on secondary metabolites and their metabolism, only points corresponding to secondary metabolites and their related genes, as identified by the KEGG database, were retained, while other points were removed. To predict the relationship between groups of metabolites and groups of DEGs, the top 20 items on each joint component with the highest loading values were selected and considered to be related. Bar charts and Circos plots were generated to visually depict these relationships and draw conclusions.

### 4.7. Statistics and Data Visualization

All statistical analyses and data visualization in this research were performed using the R programming language (version 4.3.1, if not specified) and various packages, including ‘ggplot2’ [62], ‘reshape2’ [63], ‘dplyr’, ‘tidyr’, ‘stringr’ (https://stringr.tidyverse.org, accessed 15 November 2023), ‘RColorBrewer’, ‘ggh4x’, ‘ggpubr’, ‘VennDiagram’, ‘pheatmap’, ‘ggrepel’, ‘rjson’, ‘OmicsPLS’ [61], ‘KEGGREST’, ‘DiagrammeR’, and ‘circlize’ [64].

## 5. Conclusions

Soilless cultivation is becoming increasingly popular for providing essential nutrients to greenhouse crops [65]. This study reveals those cultivating cucumbers in a coil with a moderate electrical conductivity (EC) level of 5 dS·m^−1^ enhances fruit quality. We discovered that primary metabolites (such as cellulose and amino acids) and secondary metabolites (like rutin and cucurbitacin B) were most abundant at this EC level. Transcriptome profiling on cucumber leaves revealed that EC significantly influences the expression of genes associated with photosynthesis, various anabolic processes, and membrane transport. Further O2PLS analysis identified a set of genes involved in the synthesis of amino acids, polysaccharides, and many secondary metabolites, all of which contribute to improving cucumber fruit quality. While this study found favorable responses at an EC level around 5 dS·m^−1^, it is important to note that nutrient solution concentrations for greenhouse cucumber production are generally maintained around 3 dS·m^−1^. Further studies are needed to explore the salinity response within the intermediate range of 3 dS·m^−1^ to 5 dS·m^−1^. Overall, these results suggest optimal EC conditions in soilless coir cultivation can promote desirable metabolic and genetic changes, enhancing cucumber fruit quality.

## Data Availability

The RNAseq data are available in the GEO of the National Center for Biotechnology Information (NCBI) with the accession number PRJNA1164453.

## Supplementary Materials

The following supporting information can be downloaded at: https://www.mdpi.com/article/10.3390/ijms252211864/s1.
