# Supplementary material for: The Impact of Nutrient Solution Electrical Conductivity on Leaf Transcriptome Contributing to the Fruit Quality of Cucumber Grown in Coir Cultivation
Source: Int J Mol Sci. 2024 Nov 5;25(22):11864. doi: 10.3390/ijms252211864 (PMC11593475; doi:10.3390/ijms252211864)
Supplement: Supplementary file 1 [file ijms-25-11864-s001.zip › ijms-3268003-supplementary.pdf]

The impact of nutrient solution electrical conductivity on leaf transcriptome contributing to fruit quality of cucumber grown in coir cultivation

Lizhong He[a,1], Wentao Xu[b,1], Dongke Zhou, Jun Yan[a], Haijun Jin[a], Hongmei Zhang[a], Jiawei Cui[a], Chen Miao[a], Yongxue Zhang[a], Qiang Zhou[a], Jizhu Yu[a], Xiang Yu[b,*] Xiaotao Ding[a,*]

[a]Shanghai Key Lab of Protected Horticultural Technology, Horticultural Research Institute, Shanghai Academy of Agricultural Sciences, Shanghai 201403, China

[b]Joint International Research Laboratory of Metabolic & Developmental Sciences, School of Life Sciences and Biotechnology, Shanghai Jiao Tong University, Shanghai, China

1. These authors contributed equally to this study

* Corresponding authors. *E-mail address:* yuxiang2021@sjtu.edu.cn *and dingxiaotao@saas.sh.cn*

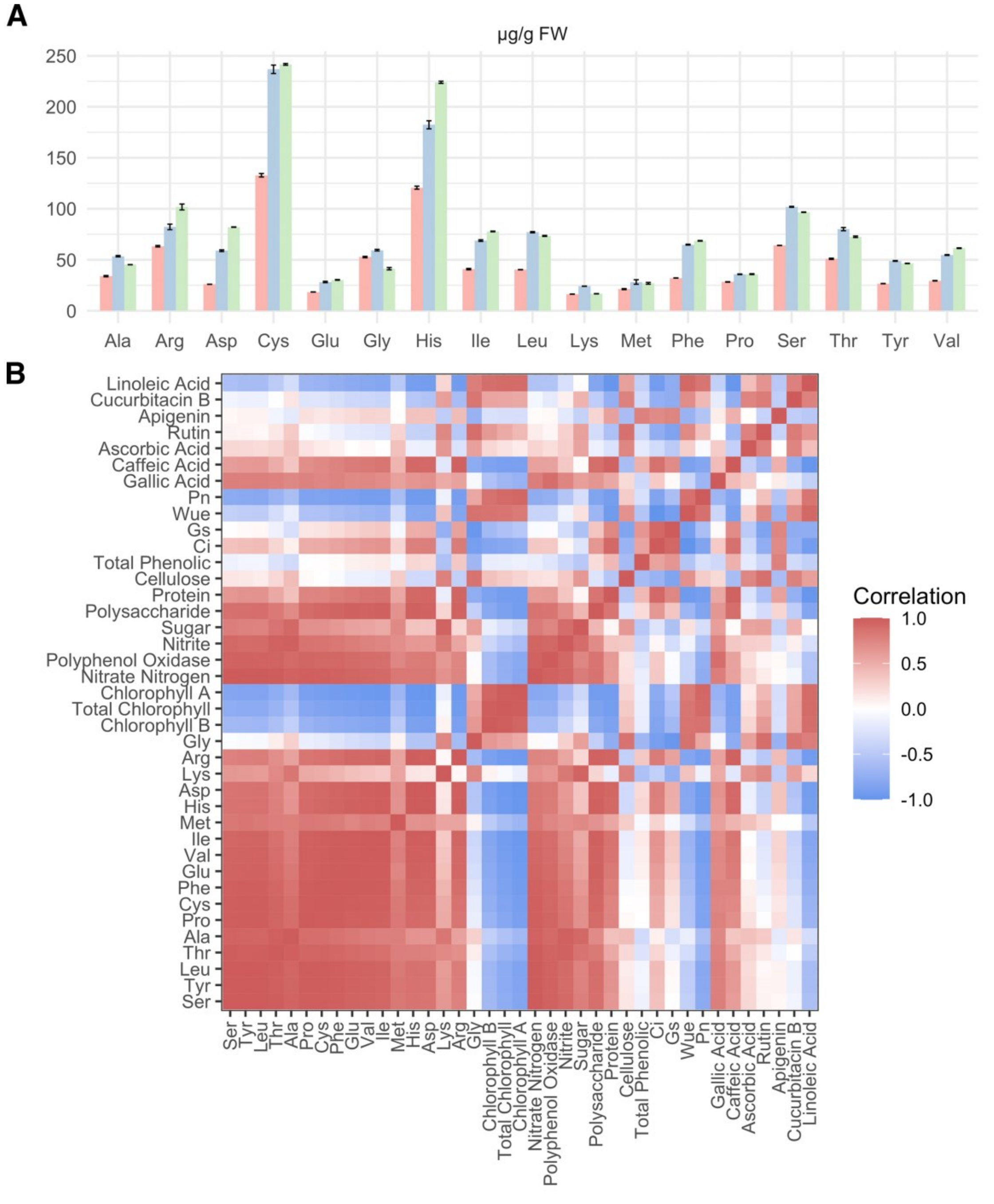

Figure S1. Metabolite abundance and correlation of *Cucumis sativa* under different conditions. (A) Abundance of some amino acids. (B) Pairwise correlation coefficient of all metabolites being investigated.

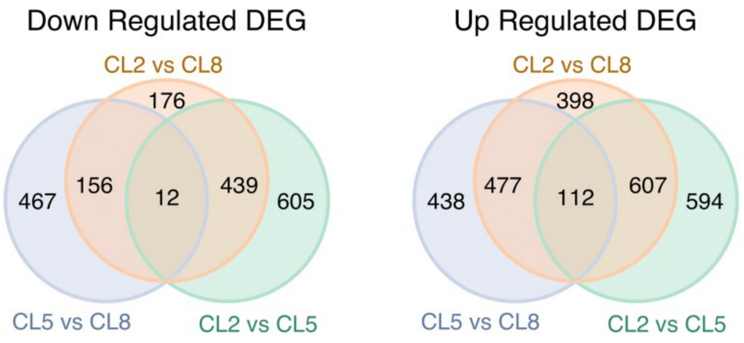

Figure S2. Venn diagram on the distribution of up- and down- regulated DEGs.

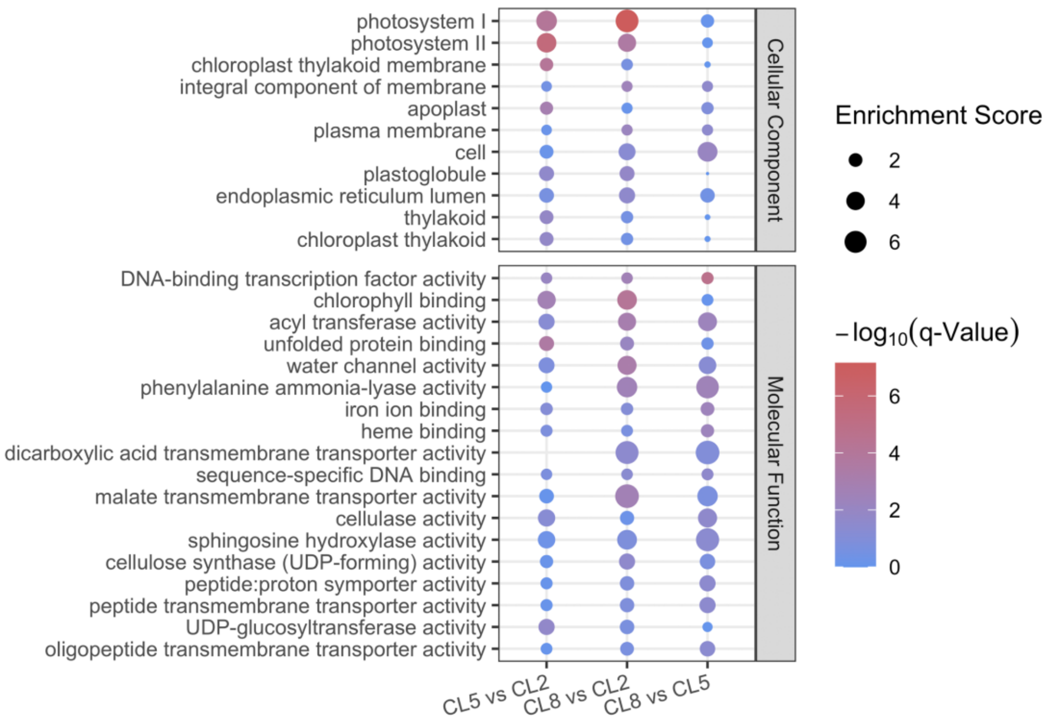

Figure S3. GO enrichment analysis for differentially expressed genes between any two groups.

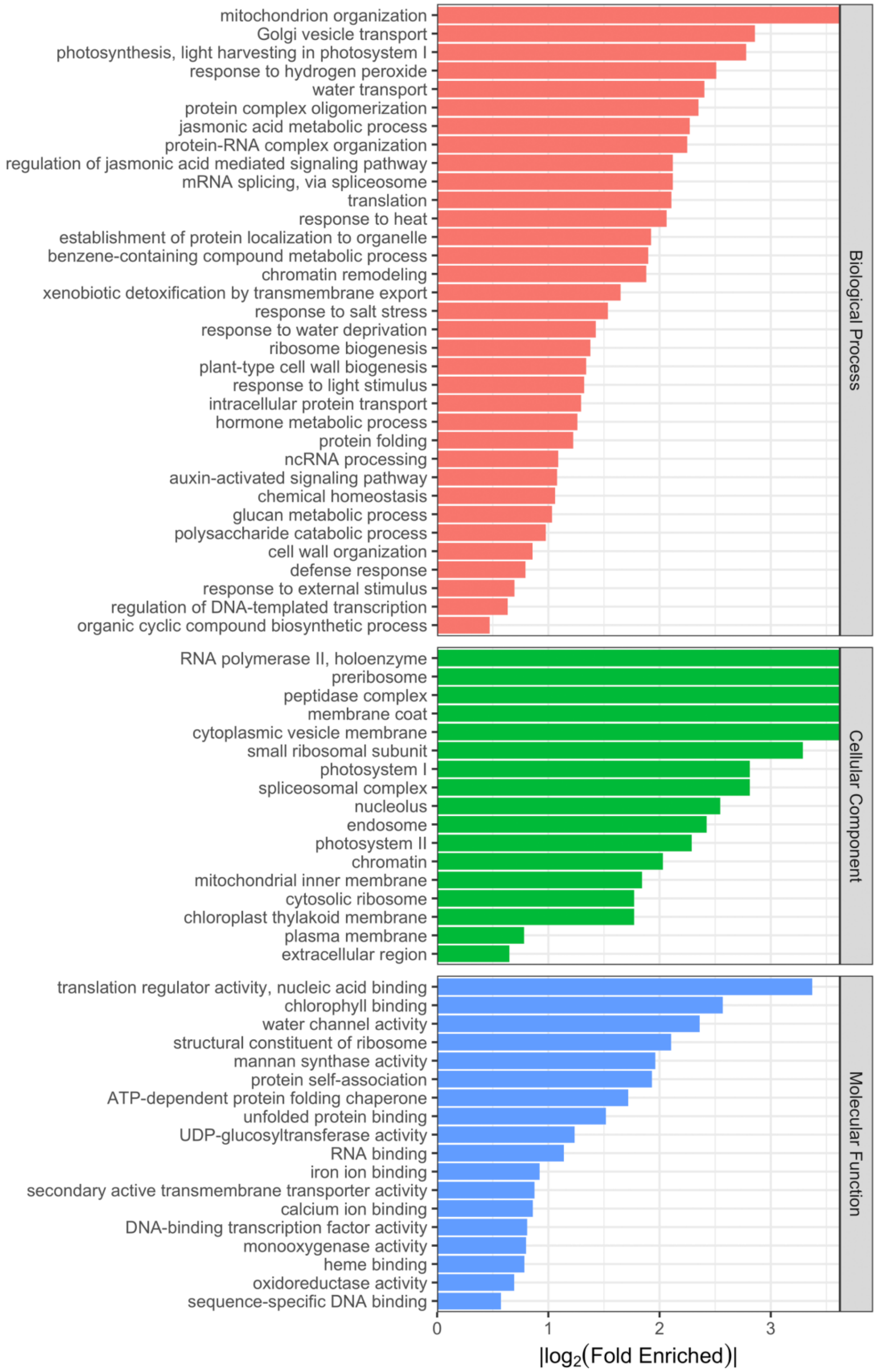

Figure S4. GO enrichment analysis for the union of DEGs. Terms with FDR < 0.05 are listed and ranked by enriched fold change.

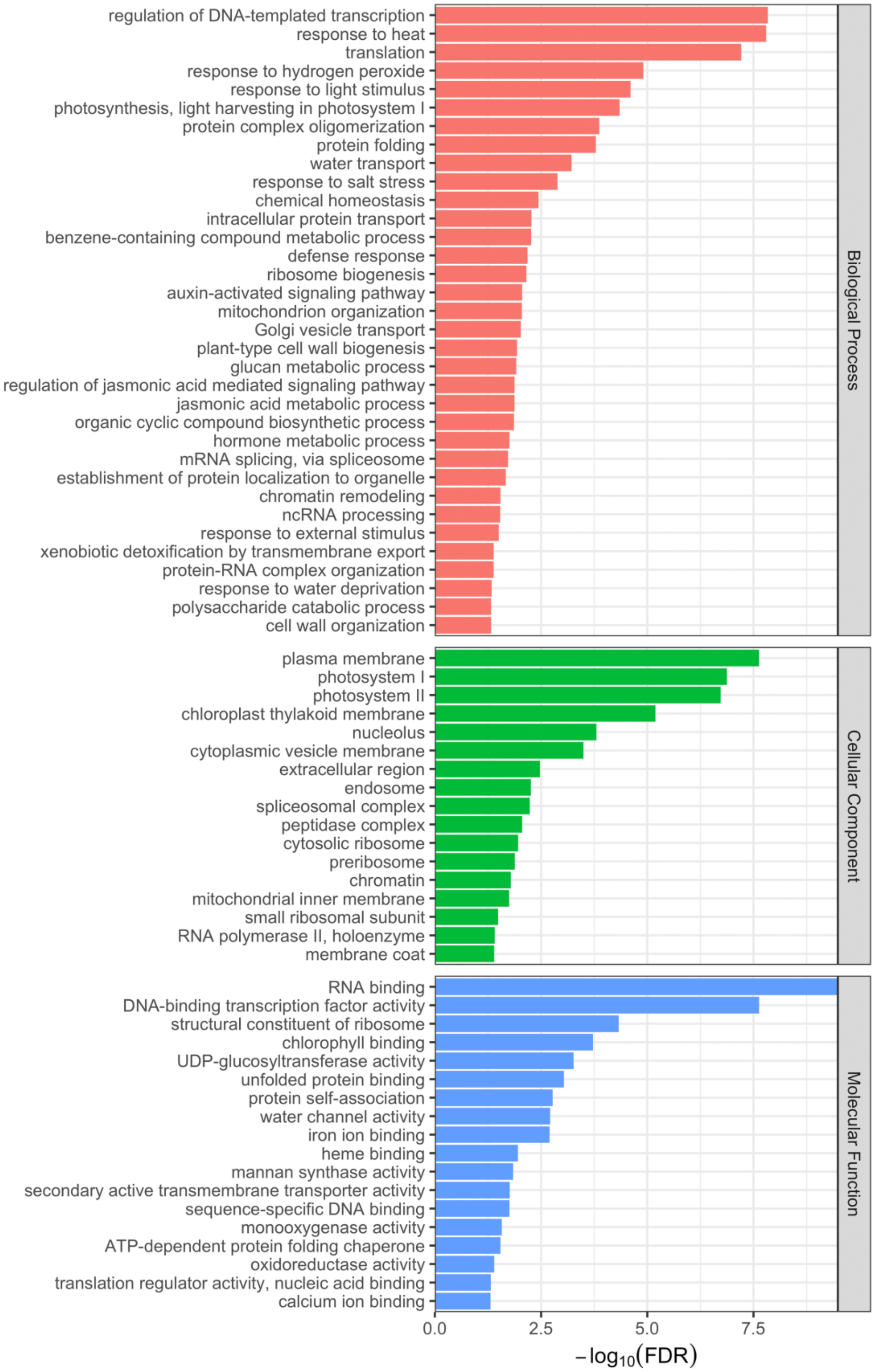

Figure S5. GO enrichment analysis for union of DEGs. Terms with FDR < 0.01 are listed and ranked by enriched -$\log_{10}$(FDR).
